# Contributions of natural killer cells to the immune response against *Plasmodium*

**DOI:** 10.1186/s12936-019-2953-1

**Published:** 2019-09-18

**Authors:** Kristina S. Burrack, Geoffrey T. Hart, Sara E. Hamilton

**Affiliations:** 1 Department of Medicine, Hennepin Healthcare Research Institute, Minneapolis, MN 55415 USA; 2 Center for Immunology, Department of Medicine, Division of Infectious Disease and International Medicine, University of Minnesota, Minneapolis, MN 55455 USA; 3 Center for Immunology, Department of Laboratory Medicine and Pathology, University of Minnesota, Minneapolis, MN 55414 USA

**Keywords:** Malaria, NK cells, Cytotoxicity, Cytokines

## Abstract

Natural killer (NK) cells are important innate effector cells that are well described in their ability to kill virally-infected cells and tumors. However, there is increasing appreciation for the role of NK cells in the control of other pathogens, including intracellular parasites such as *Plasmodium*, the cause of malaria. NK cells may be beneficial during the early phase of *Plasmodium* infection—prior to the activation and expansion of antigen-specific T cells—through cooperation with myeloid cells to produce inflammatory cytokines like IFNγ. Recent work has defined how *Plasmodium* can activate NK cells to respond with natural cytotoxicity, and inhibit the growth of parasites via antibody-dependent cellular cytotoxicity mechanisms (ADCC). A specialized subset of adaptive NK cells that are negative for the Fc receptor γ chain have enhanced ADCC function and correlate with protection from malaria. Additionally, production of the regulatory cytokine IL-10 by NK cells prevents overt pathology and death during experimental cerebral malaria. Now that conditional NK cell mouse models have been developed, previous studies need to be reevaluated in the context of what is now known about other immune populations with similarity to NK cells (i.e., NKT cells and type I innate lymphoid cells). This brief review summarizes recent findings which support the potentially beneficial roles of NK cells during *Plasmodium* infection in mice and humans. Also highlighted are how the actions of NK cells can be explored using new experimental strategies, and the potential to harness NK cell function in vaccination regimens.

## Background

Natural killer (NK) cells are lymphocytes of the innate immune system that participate in early defense against foreign cells and autologous cells undergoing various forms of stress, such as microbial infection or tumour transformation [1, 2]. NK cells are derived from a common lymphoid progenitor and develop primarily in the bone marrow [2]. Under homeostatic conditions, NK cells are abundant in blood, spleen, bone marrow, and liver, but they can migrate to inflamed or infected tissues in response to chemoattractants [3]. NK cells recognize Human Leukocyte Antigen (HLA) class I molecules via Killer Immunoglobulin Receptors (KIRs) that predominantly deliver signals that suppress, rather than activate NK cell function [2]. NK cell activation decisions are based on the balance of engaged inhibitory or activating receptors such as KIR, natural cytotoxicity receptors (NCRs), DNAX accessory molecule-1 (DNAM-1) and NK group 2 member D (NKG2D) [2, 4]. When inhibitory signals are reduced or absent, activating NK cell receptor ligation results in NK cell triggering and target cell death; this is referred to as natural cytotoxicity. In addition to natural cytotoxicity, NK cells can cooperate with the adaptive immune system by performing antibody-dependent cellular cytotoxicity (ADCC) upon recognition of opsonized targets via the Fc receptor—FcRγIIIa (CD16). Lastly, NK cells have the ability to induce apoptosis in target cells through Fas Ligand (FasL) and tumour necrosis factor-related apoptosis-inducing ligand (TRAIL) [5, 6]. Thus, NK cells exhibit a wide variety of methods to recognize and attack cells that are a danger to the host.

NK cells also respond to cytokine signals delivered by other immune cells, namely IL-2, IL-15, IL-18, and IL-12 [7]. Exposure allows NK cells to augment immune responses through rapid and robust production of pro-inflammatory cytokines, including interferon-gamma (IFN-γ) and tumour necrosis factor (TNF), among others. Much work has focused on this bolus of cytokine production which serves to amplify immune responses, particularly in the early hours after infection. However, NK cells may also contribute to overproduction of proinflammatory cytokines, which can result in immunopathology [8]. Perhaps to temper this danger, NK cells can also produce the anti-inflammatory cytokine IL-10, which limits immune responses [9–12]. Variation in the clinical symptoms associated with *Plasmodium* infection in humans, along with diverse characteristics in specific mouse models of infection, have made defining the protective immune response challenging. There is still not a consensus on whether NK cells are overall more harmful, helpful, or inconsequential to the immune response to *Plasmodium* (see review by Wolf et al. [13]). However, several recent studies have started to gain a better understanding of the mechanisms by which NK cells are activated during malaria infection and the downstream consequences of their activation. Here, findings are highlighted that relate to the potentially beneficial actions of NK cells during *Plasmodium* infection in mice and humans. These studies justify further evaluation of NK cells in the context of malaria disease.

### NK cells during liver stage infection

**Fig. 1:**
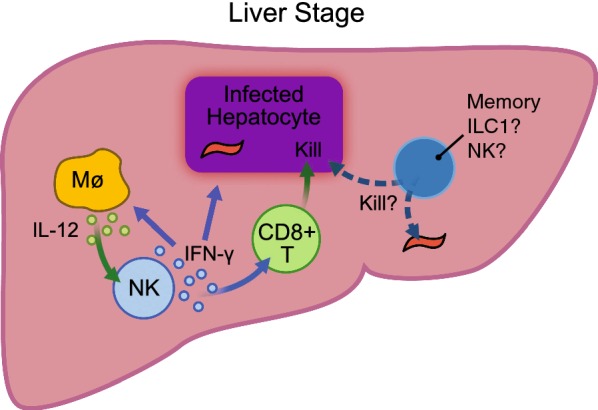
Liver stage infection or sporozoite immunization. During the liver stage, NK cells may respond to IL-12 stimulation by making IFN-γ. This could serve to augment the immune response directed against infected hepatocytes. A plausible, but unproven mechanism is that NK cells may also kill infected hepatocytes or sporozoites

After being bitten by a mosquito carrying *Plasmodium* parasites, a low number of sporozoites (on the order of 1–25) are transmitted [14]. The sporozoites travel through the blood stream to the liver and infect a small number of hepatocytes, where they replicate and differentiate into merozoites. Human trials with the RTS,S vaccine indicate that antibody against circumsporozoite protein (CSP) and CD4^+^ T cell responses serve as good correlates of protection [15]. CD8^+^ T cells are also implicated as critical effector cells in protection against pre-erythrocytic stage malaria [16, 17]. To obtain robust responses, CD8^+^ T cells are primed by liver-infiltrating CD11c^+^ cells that acquire *Plasmodium* antigens, traffic to the liver draining lymph nodes, and then present peptides to naive T cells [18]. NK and NKT cells are also abundant in the liver, and they are early producers of IFN-γ, which is an important effector molecule that could conceivably contribute to the activation of immune cells and indirectly lead to destruction of parasite-infected hepatocytes (Fig. 1) [19, 20].

Observational studies in humans have suggested that NK cells contribute to immunity against malaria during the liver stage of disease. However, human challenge studies are limited to showing that infection and increased protection correlated with decreased frequency and number of NK cells in the blood of subjects [21–23]. Although it is tempting to speculate that this could be due to increased trafficking to the infected liver, this is difficult to address experimentally in humans. Enhanced IFNγ production by human NK cells has been observed after RTS,S/AS01 malaria vaccination [20]. These improved responses could be due to either indirect activation of NK cells by cytokines or potentially, cognate antigen recognition. Regardless of the mechanism, NK cells in the liver might be sufficiently stimulated by vaccination to meaningfully contribute indirectly or directly to protective immune responses against *Plasmodium* [24].

Studies in mice have shown that NK and NKT cells both increase in number in the liver and produce increased amounts of IFNγ and TNF in response to *Plasmodium yoelii* infection [25, 26], which could be important to dampen the growth of schizonts in the liver and amplify the early immune response. Early work investigating the protective mechanisms of radiation-attenuated sporozoites used in vivo antibody depletion to conclude that, in addition to CD8^+^ T cells, NK cells are required for vaccine-induced protection against *P. yoelii* challenge [27]. This was proposed to be the result of IL-12 activation of NK cells, which in-turn made IFNγ. Additionally, using CD1d^−/−^ mice, Miller et al. showed that NKT cells play a significant role in lowering liver parasite burden [26]. Future work can reevaluate the importance of NK cell-specific cytokine production using new, more specific technical approaches. For example, the use of *Nkp46*^*iCre*^ mice [28] or NKp46-CreERT2 mice [29] bred with mice containing loxP sites flanking effector molecules (IFNγ, TNF) [30] would help to bolster or disprove the case that NK cell-derived cytokine production is important for *Plasmodium* control.

Overall, additional studies are required, in both humans and mice, to determine the exact role, if any, NK cells play in the liver during *Plasmodium* infection. The presence of additional liver populations including resident NK cells and type 1 innate lymphoid cells (ILC1s) further complicates assignment of individual immune cell contributions to protection from the liver stage of disease. ILC1s are CD49a^+^ CD49b^−^ CXCR6^+^ T-bet^+^ Eomes^−^ CD127^lo/−^ and are predominantly tissue-resident [31]. Conventional NK cells are largely recirculating and can be identified based on the following phenotype: CD49a^−^ CD49b^+^ CXCR6^−^ T-bet^+^ Eomes^+^ [31, 32].

To date, the contribution of NK-related cell types to protection in the liver has often relied on antibody depletion using shared markers (i.e., NK1.1). Thus, future experiments will need to precisely determine the individual impact of conventional NK cells and other similar immune cell types to pre-erythrocytic immunity.

### NK cells during blood stage infection

Merozoites released from hepatocytes initiate the blood-stage of infection. In the bloodstream, free merozoites invade RBCs beginning a 48-h cycle of maturation followed by RBC rupture, and additional release of merozoites. Release of mature parasites from infected RBCs (iRBCs) results in symptomatic disease characterized by fever, headache, and lethargy. The most vulnerable are young children and pregnant women; severe disease takes the form of anaemia, placental disruption and miscarriage, acute respiratory distress, and cerebral manifestations [33]. With age and re-exposure, the risk of malaria goes down, and although hosts continue to carry parasite burdens, they usually do so without the associated symptoms.

#### Non-severe infection- human and humanized mouse studies

During uncomplicated malaria in *Plasmodium falciparum*-infected Kenyans, NK cell frequencies (CD56^+^CD3^−^) were reduced relative to asymptomatic and aparasitaemic people [34]. Similar results were found in a recent study of Mali subjects, but this report additionally showed that the CD56^dim^ subset was specifically reduced during malaria episodes, whereas the CD56^bright^ population was increased [35]. Other studies have investigated the potential protective role of NK cells during blood stage *Plasmodium* infection. In both mouse and human studies, IFNγ has been associated with control of parasitaemia, protection from malaria, and delayed reinfection [36–40]. NK cells rapidly produce IFNγ in the presence of many pathogens, including *Plasmodium* [37, 41]. However, in vitro assays indicate that IFNγ production against *Plasmodium* requires direct contact between NK cells and iRBCs and a cellular source of IL-12 and IL-18, such as monocytes or DCs [42–44].

Evidence for direct NK cell natural cytotoxicity toward *P. falciparum*-infected erythrocytes was first demonstrated by Orago and colleagues via in vitro chromium release assay [34]. This was subsequently reproduced by other studies demonstrating antibody-independent lysis of infected red blood cells by NK cells, however the percent of natural cytotoxicity responders in studies is variable [45–47]. When PBMCs from malaria-naïve individuals are incubated with iRBCs, NK cells have been shown to increase expression of the cytotoxic molecules perforin, granzyme A, and CD107a (LAMP-1) [41]. It was subsequently shown that NK cells form stable conjugates with iRBCs leading to cytoskeletal actin rearrangement [41]. A recent study showed that human NK cells can perform natural cytotoxicity in vitro, and utilized a humanized mouse model of *Plasmodium* infection (reviewed in [48]) to demonstrate that NK cells were critical for the control of parasitaemia in vivo [49]. Humanized mouse models have several limitations; in this study, the mice exhibited low parasitaemia relative to what is observed in humans, and the ligand/receptor combinations required for NK cell recognition of iRBCs have not been fully elucidated. It has been shown that antibody-mediated inhibition of NKp30 and to a lesser extent NKp46 (both natural cytotoxicity receptors) reduced lysis of iRBCs [46]. Preincubation of NK cells with a peptide from the *P. falciparum* erythrocyte membrane protein-1 (*Pf*EMP-1) also reduced lysis, but other *Plasmodium* antigens did not have an effect [46]. These data suggest that NK cell natural cytotoxicity receptors can directly recognize *P. falciparum* PfEMP-1 proteins on iRBCs to mediate lysis. In a separate study, Saito and colleagues found that a subset of RIFIN proteins, which are expressed on the surface of iRBCs, bound to the inhibitory receptors LILRB1 or LAIR1 on NK cells and reduced NK cell cytotoxicity [50]. A recent report suggests that microvesicles from iRBCs can fuse with NK cells in vitro and cause activation and a reduction in parasitaemia [51]. The authors found that the innate receptor MDA5, which can bind uncapped RNA, could be induced in “iRBC responsive” NK cells, and that genetic knockdown of MDA5 resulted in the loss of parasitaemia control in vitro, suggesting that parasite RNA in the microvesicles stimulated NK cells to kill iRBCs. Ye et al. also showed that stimulation of “iRBC non-responsive” NK cells with a MDA5 agonist induced activation and improved control of parasitaemia, providing evidence of a molecular basis for variation in human NK cell responses to malaria [51]. However, the proportion of people with “responder” vs. “non-responder” NK cell phenotypes was not reported.

**Fig. 2:**
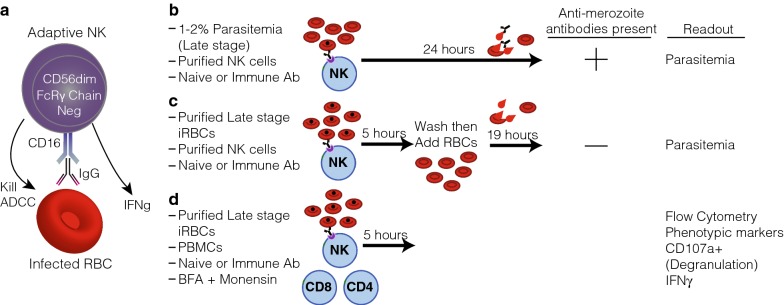
Assays to look at Natural Killer cell function. **a** Model of the subset of adaptive NK cells that lack Fc receptor γ chain. These cells are particularly skilled at degranulating and producing IFNγ that can help activate the immune response to *Plasmodium.* **b** Growth inhibition antibody dependent cellular cytotoxicity assay (Alternative GI-ADCC assay). Synchronized late stage parasite culture is incubated with or without natural killer cells and with or without immune plasma or antibodies. In this assay, the NK cells can kill the infected RBCs but the antibody is still there to allow for growth inhibition via neutralizing activity against merozoites. The resulting inhibition can then be quantified in all groups and compared by looking at parasitaemia. **c** Growth inhibition antibody dependent cellular cytotoxicity assay (GI-ADCC assay). In this assay late stage purified (> 95% pure) infected RBCs are mixed with NK cells with or without immune plasma or antibodies. After 5 h the antibodies are washed out, then uninfected RBCs are added at 100 fold excess. The infected RBCs then rupture and the resulting parasitaemia the next day is used to assess growth inhibition. **d** Functional analysis of NK cells in mixed PBMCs. PBMCs are incubated at a 1:1 ratio with late stage purified infected RBCs then immune or naive plasma is added. The phenotype and ADCC function is then assessed via surface markers, CD107a as a marker for degranulation and IFNγ by intracellular cytokine staining

A recent study showed that NK cell-mediated cytotoxicity required antibody; that is, NK cells killed iRBCs via ADCC and inhibited the growth of *Plasmodium* in vitro [52] **(**Fig. 2a). In this study the destruction of iRBCs was highly specific, as uninfected RBCs in the same culture were not lysed, and killing was dependent on Fc receptors. It was also shown that a monoclonal antibody with modifications to the Fc portion of the antibody—making it unable to bind to the Fc receptor CD16—abrogated the ADCC activity [53]. The in vitro growth inhibition assay was performed using two different approaches. The first approach added NK cells at a 3:1 effector (NK) to target (late stage infected RBC) ratio with naïve (USA) serum or hyperimmune serum (pooled Mali adult plasma containing antibodies against both the iRBC surface and merozoites), then let the culture grow. The addition of NK cells increased growth inhibition significantly over hyperimmune plasma alone (averaging 80% vs. 20% respectively), however anti-merozoite antibodies could account for some of the growth inhibition (Fig. 2b) [52]. In addition to this assay, purified late stage infected RBCs were incubated with NK cells at a 3:1 (Effector:Target) ratio for 5 h with USA or Mali plasma, the cells were washed to get rid of any antibodies, and fresh uninfected RBCs were added (Fig. 2c). Growth inhibition was still observed in this assay in the absence of anti-merozoite antibodies. The contribution of complement was also removed by using plasma free system and purified IgG from the same naïve or immune plasma [52]. In total, these assays implicate NK cell-mediated ADCC as a potential mechanism of acquired immunity that effectively limits *Plasmodium* growth. Another study also found that in the presence of antibody FasL may be involved as a killing mechanism [45], however a comprehensive assessment of how growth is inhibited is still needed.

Recently, it was demonstrated that adaptive NK cells correlated with protective malaria phenotypes, including decreased parasitaemia and reduced symptoms [35]. Adaptive NK cells are long-lived and have enhanced functionality, but in the past were studied only in the context of human cytomegalovirus (HCMV) and human immunodeficiency virus (HIV) [54, 55]. In the context of *Plasmodium* infection, adaptive NK cells, which lacked Fc receptor γ chain [55, 56], performed enhanced ADCC function for both degranulation and IFN-γ production in an in vitro PBMC assay (Fig. 2d) [35]. Additionally, subjects that displayed increased frequencies of promyelocytic leukemia zinc finger protein (PLZF) negative adaptive NK cells (which lack a key transcription factor for Fc receptor γ chain expression) in the current season were significantly protected from malaria symptoms the following malaria season [35]. Increased frequencies of PLZF negative adaptive NK cells also correlated with reduced parasitaemia and was found to be independent of age [35]. These studies provide the first evidence that one function of protective antibodies may be to “tag” iRBCs for removal by NK cells, leading to reduced parasitaemia and disease symptoms. The frequency of adaptive NK cells in the Malian cohort was 40% of the NK cells and most of the subjects were CMV positive. In non-malaria exposed subjects that are HCMV positive, frequencies of adaptive NK cells are generally not this high (< 10%) [55, 56]. It is unclear if HCMV co-infection with malaria further increases adaptive NK cell frequencies. Because mouse NK cells do not perform ADCC well [57, 58], additional studies using human NK cell and humanized mouse systems are needed to further investigate this mechanism. Since it was shown that NK cells killed iRBCs but not merozoites via ADCC [35], the data suggest that surface-expressed iRBC proteins may be good targets for vaccines designed to enhance NK cell function. These data also suggest that promoting the generation of antibodies that bind the CD16 Fc receptor—IgG1 and IgG3 isotypes, which are also complement-fixing antibodies—could benefit parasite clearance.

#### Non-severe infection- mouse studies

*Plasmodium falciparum* is restricted to humans, but several *Plasmodium* strains can infect mice and replicate features of human malaria disease. No one model recapitulates all features of human malaria disease states; however, depending on the strain of parasite and the genetic background of the mouse strain used, a wide range of outcomes can be mechanistically investigated, from parasitaemia to cerebral malaria [59]. Using *Plasmodium chabaudi* or *P. yoelii* 17X infection as models of blood stage infection, NK cell frequencies increase [60] as does their production of IFNγ and TNF [40]. Depletion of NK cells with anti-NK1.1 or anti-asialo GM1 Ab resulted in increased mortality, decreased IFNγ, and either slightly increased or no change in parasitaemia levels [22, 61, 62]. These data suggest that NK cells may contribute to control of parasitaemia and/or disease development during *P.c.* and *P.y.* infection models in mice, but the approaches used are not specific enough to definitively say that NK cells play a fundamental role in protection.

#### Severe infection

**Fig. 3:**
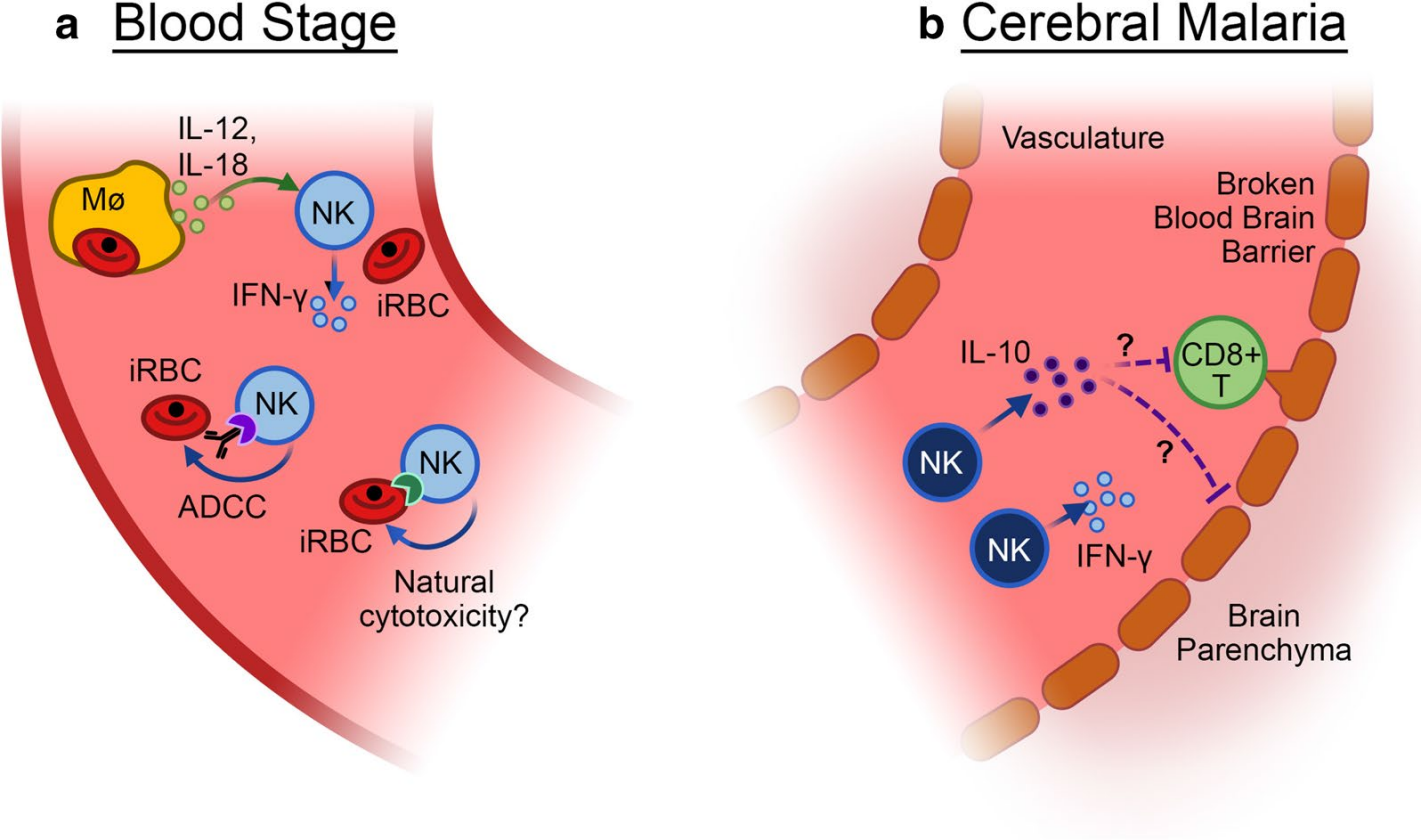
Blood stage infection. **a** During the blood stage of *Plasmodium* infection, NK cells reduce parasitaemia via production of IFN-γ and direct killing of iRBCs, **b** During experimental cerebral malaria, IFN-γ-producing NK cells may promote inflammation in the brain. However, with appropriate stimulation (e.g. IL-15 cytokine complexes), NK cells can produce IL-10 and prevent the oedema and pathology associated with ECM through effects on CD8^+^ T cells and/or brain endothelium

One severe result of blood stage *P. falciparum* infection is the development of neurological complications known as cerebral malaria (CM). CM mostly afflicts children under the age of five in sub-Saharan Africa and can be fatal [63]. Furthermore, over 25% of children that survive CM exhibit long-term cognitive impairment [64, 65]. There are no adjunctive therapies available for CM patients. CM pathogenesis is generally considered to be driven by two distinct processes: sequestration of iRBCs to vascular endothelium and inflammation [66, 67] (Fig. 3). The sequestration and excessive inflammation lead to the loss of blood brain barrier integrity, ultimately resulting in oedema [68–70].

Infiltration of inflammatory immune cells into the brains of CM patients has not been extensively investigated due to the difficult nature of obtaining samples. A study from three decades ago found that CM patients had significantly reduced NK cell cytotoxicity against K562 cells compared with healthy controls or patients with uncomplicated *P. falciparum* infection [71]. A recent study compared the ability of NK cells purified from patients with uncomplicated malaria to those with severe malaria to control parasite growth in vitro; although the sample size was relatively small, the data suggest that NK cells from patients with severe malaria disease are less capable of controlling *P. falciparum* growth in vitro [51]. This could be due to immune suppression during severe disease or a ‘hyper-immune’ response that ultimately leads to NK cell functional exhaustion [72–74].

In contrast to the relative scarcity of studies on the role of NK cells during human CM, numerous studies have investigated the role of NK cells in the mouse model of CM, known as experimental cerebral malaria (ECM). The ECM mouse model replicates the neurological symptoms, parasite accumulation, and leukocyte infiltration observed in children with CM, including the induction of brain oedema and brain stem death as a predictor of severe disease [75–77]. Numerous studies in both mice and humans have demonstrated that an excessive pro-inflammatory response results in severe immunopathology during malaria [78–80]. IFNγ—which can be secreted in large amounts by NK cells—is a critical mediator of immune-mediated damage during ECM [81, 82]. An initial report using anti-NK1.1 Ab to deplete NK cells suggested that NK cells play no role in ECM [80]. More recent reports using anti-Asialo GM1 Ab suggested that NK cells actively contribute to ECM [83, 84]. This treatment reduced CD8^+^ T cell priming in the spleen [84] and upregulation of CXCR3 expression [83], a chemokine receptor important for T cell trafficking to the brain. However, treatment with NK1.1 can also deplete NKT cells and innate lymphoid cells (ILCs), whereas anti-Asialo GM1 treatment has been reported to deplete macrophages, basophils, and CD8 T cells among other immune cells. Other studies investigating the natural killer complex (NKC), a genetic region of highly inked genes encoding several receptors involved in the control of NK cell function, suggest that polymorphisms within the NKC modulate control susceptibility to the development of ECM and the type of immune response (TH1 vs. TH2) that unfolds [85, 86]. These data as a whole suggest that the pro-inflammatory nature of NK cells could contribute to immune-mediated damage during ECM, but development of additional NK cell-specific depletion methods or use of adoptive transfer approaches [9, 87] need to be employed in future studies to definitively identify any pathological role of NK cells during ECM.

In contrast to a potential pathologic role for NK cells, it was recently shown that NK cells can be induced to produce IL-10, which inhibited the pathologic CD8^+^ T cell response and protected mice from ECM [9]. There is evidence in other disease models, including from other protozoan infections, that NK cells produce IL-10 [11, 12, 88], similar to CD4^+^ T cells that can produce both IFNγ and IL-10. It was also demonstrated that stimulation of NK cells with IL-15 cytokine complexes induced this “regulatory” NK cell phenotype. Additionally, primary human NK cells produced IL-10 following culture with IL-15 and IL-21 and increase production further with the addition of IL-12 [9]. Further studies are needed to determine if IL-10-producing NK cells contribute to the healthy resolution (or prevention) of inflammation during malaria disease in humans.

Other severe complications of *Plasmodium* infection include placental malaria and severe malaria anaemia (SMA). Placental malaria can result in placental disruption and lead to miscarriages, still birth, low birth rate, and in some cases death of the pregnant woman [89]. It is estimated that 25% of pregnant women in endemic areas are infected with *Plasmodium*, and that this is responsible for 20% of maternal deaths [89]. During this disease, iRBCs express a PFEMP1 protein called Var2CSA that facilitates adherence to chondroitin sulfate A expressed on the placenta [90, 91]. This infiltration and adherence of iRBC to the placenta is believed to be responsible for the observed clinical complications, however the mechanism of disruption is still unclear. Var2CSA is a large and variable gene however its amino acid variation is around 75–85% found in clinical isolates [89]. Women can become immune to placental malaria during successive pregnancies, showing that effective immune protection can be achieved (76, 77). It is unknown if NK cells provide protection against Var2CSA + infected RBCs through natural cytotoxicity or ADCC. Very few studies have investigated NK cells in the placenta and in the blood at various timepoints [92, 93]. It is known that specialized NK cells in the uterus are necessary for angiogenesis of the placenta that occurs mainly in the first trimester and that NK cells represent 70% of all leukocytes found in the decidua [94]. Furthermore, elevated numbers of IFNγ-producing NK cells and T cells in the placenta is associated with protection [95]. Severe malaria anaemia is characterized by haemoglobin levels less than 5 g/dl, poor erythrocytic output from the bone marrow, and often enlarged spleen [96]. SMA typically occurs in areas that have high exposure rates to *Plasmodium* or are genetically susceptible to the disease. FcγRIIIa is the lone Fc receptor expressed on NK cells that enables ADCC, and polymorphisms in FcγRIIIa are associated with SMA [97]. The cell responsible for this association is unknown, but lack of function mutations correlate with poorer outcomes. Whether or not NK cells in these subjects lack effector function has not been explored. In addition, the subset(s) of NK cells associated with placenta malaria or SMA are also unknown. Overall, there is a need for additional studies to indicate if there is a role for NK cells during placental malaria and SMA.

## Conclusions

Although increased efforts toward prevention of malaria spread have resulted in significant declines in disease, there is still a need for greater understanding of how an effective immune response against *Plasmodium* unfolds. Given the mounting evidence of NK cell functional diversity and participation in the immune response to *Plasmodium*, NK cells represent a new opportunity for improving resistance to malaria—be it through therapeutics or as targets of vaccination approaches [24]. This review summarizes the evidence that NK cells can contribute to the immune response in the clearance of parasites, removal of infected hepatocytes and RBCs through natural cytotoxicity and ADCC, and dampen the adaptive immune response through production of regulatory cytokines. Therefore, NK cells may serve as a critical adjustment point for enhanced immunity to *Plasmodium* while still preventing immunopathology. However, the number of studies precisely detailing the role of NK cells during different stages of *Plasmodium* infection and during non-severe and severe forms of disease is sparse. Advances in the understanding of NK cell subsets along with technical improvements should now allow for further consideration of whether NK cells can and should be targeted to improve clinical outcomes during malaria.

## Data Availability

Not applicable.

## Abbreviations

NK: natural killer cell
MHC: major histocompatibility
KIR: killer immunoglobulin-like receptors
NCRs: natural cytotoxicity receptors
DNAM-1: DNAX accessory molecule-1
NKG2D: natural killer cell group 2 member D
ADCC: antibody dependent cellular cytotoxicity
TRAIL: tumour necrosis factor-related apoptosis-inducing ligand
IFNγ: interferon gamma
TNF: tumour necrosis factor
CSP: circumsporozoite protein
ILC1s: type 1 innate lymphoid cells
RBCs: red blood cells
iRBCs: infected red blood cells
P*f*EMP-1: *P. falciparum* erythrocyte membrane protein-1
HCMV: human cytomegalovirus
HIV: human immunodeficiency virus
PLZF: promyelocytic leukaemia zinc finger protein
CM: cerebral malaria
ECM: experimental cerebral malaria
ILCs: innate lymphoid cells
SMA: severe malaria anaemia

## References

[1] Mody CH, Ogbomo H, Xiang RF, Kyei SK, Feehan D, Islam A (2019). Microbial killing by NK cells. J Leukoc Biol.

[2] Long EO, Kim HS, Liu D, Peterson ME, Rajagopalan S (2013). Controlling natural killer cell responses: integration of signals for activation and inhibition. Annu Rev Immunol.

[3] Moretta A, Bottino C, Mingari MC, Biassoni R, Moretta L (2002). What is a natural killer cell?. Nat Immunol.

[4] Moretta A, Pende D, Locatelli F, Moretta L (2009). Activating and inhibitory killer immunoglobulin-like receptors (KIR) in haploidentical hemopoietic stem cell transplantation to cure high-risk leukaemias. Clin Exp Immunol.

[5] Wallin RP, Screpanti V, Michaelsson J, Grandien A, Ljunggren HG (2003). Regulation of perforin-independent NK cell-mediated cytotoxicity. Eur J Immunol.

[6] Kayagaki N, Yamaguchi N, Nakayama M, Takeda K, Akiba H, Tsutsui H (1999). Expression and function of TNF-related apoptosis-inducing ligand on murine activated NK cells. J Immunol..

[7] Romee R, Leong JW, Fehniger TA (2014). Utilizing cytokines to function-enable human NK cells for the immunotherapy of cancer. Scientifica (Cairo)..

[8] King T, Lamb T (2015). Interferon-gamma: the Jekyll and Hyde of malaria. PLoS Pathog.

[9] Burrack KS, Huggins MA, Taras E, Dougherty P, Henzler CM, Yang R (2018). Interleukin-15 complex treatment protects mice from cerebral malaria by inducing interleukin-10-producing natural killer cells. Immunity.

[10] Clark SE, Filak HC, Guthrie BS, Schmidt RL, Jamieson A, Merkel P (2016). Bacterial manipulation of NK cell regulatory activity increases susceptibility to *Listeria monocytogenes* infection. PLoS Pathog.

[11] Perona-Wright G, Mohrs K, Szaba FM, Kummer LW, Madan R, Karp CL (2009). Systemic but not local infections elicit immunosuppressive IL-10 production by natural killer cells. Cell Host Microbe.

[12] Tarrio ML, Lee S-H, Fragoso MF, Sun H-W, Kanno Y, O’Shea JJ (2014). Proliferation conditions promote intrinsic changes in NK cells for an IL-10 response. Journal of Immunology..

[13] Wolf AS, Sherratt S, Riley EM (2017). NK cells: uncertain allies against malaria. Front Immunol..

[14] Beier JC, Davis JR, Vaughan JA, Noden BH, Beier MS (1991). Quantitation of *Plasmodium falciparum* sporozoites transmitted in vitro by experimentally infected *Anopheles gambiae* and *Anopheles stephensi*. Am J Trop Med Hyg.

[15] White MT, Bejon P, Olotu A, Griffin JT, Riley EM, Kester KE (2013). The relationship between RTS, S vaccine-induced antibodies, CD4(+) T cell responses and protection against *Plasmodium falciparum* infection. PLoS ONE.

[16] Crompton PD, Moebius J, Portugal S, Waisberg M, Hart G, Garver LS (2014). Malaria immunity in man and mosquito: insights into unsolved mysteries of a deadly infectious disease. Annu Rev Immunol.

[17] Kurup SP, Butler NS, Harty JT (2019). T cell-mediated immunity to malaria. Nat Rev Immunol.

[18] Kurup SP, Anthony SM, Hancox LS, Vijay R, Pewe LL, Moioffer SJ (2019). Monocyte-derived CD11c(+) cells acquire Plasmodium from hepatocytes to prime CD8 T cell immunity to liver-stage malaria. Cell Host Microbe.

[19] Stegmann KA, De Souza JB, Riley EM (2015). IL-18-induced expression of high-affinity IL-2R on murine NK cells is essential for NK-cell IFN-gamma production during murine *Plasmodium yoelii* infection. Eur J Immunol.

[20] Horowitz A, Hafalla JC, King E, Lusingu J, Dekker D, Leach A (2012). Antigen-specific IL-2 secretion correlates with NK cell responses after immunization of Tanzanian children with the RTS, S/AS01 malaria vaccine. J Immunol..

[21] Mpina M, Maurice NJ, Yajima M, Slichter CK, Miller HW, Dutta M (2017). Controlled human malaria infection leads to long-lasting changes in innate and innate-like lymphocyte populations. J Immunol..

[22] Ng SS, Souza-Fonseca-Guimaraes F, de Rivera FL, Amante FH, Kumar R, Gao Y (2018). Rapid loss of group 1 innate lymphoid cells during blood stage Plasmodium infection. Clin Transl Immunol..

[23] Kazmin D, Nakaya HI, Lee EK, Johnson MJ, van der Most R, van den Berg RA (2017). Systems analysis of protective immune responses to RTS, S malaria vaccination in humans. Proc Natl Acad Sci USA.

[24] Wagstaffe HR, Mooney JP, Riley EM, Goodier MR (2018). Vaccinating for natural killer cell effector functions. Clin Transl Immunol..

[25] Roland J, Soulard V, Sellier C, Drapier AM, Di Santo JP, Cazenave PA (2006). NK cell responses to Plasmodium infection and control of intrahepatic parasite development. J Immunol..

[26] Miller JL, Sack BK, Baldwin M, Vaughan AM, Kappe SH (2014). Interferon-mediated innate immune responses against malaria parasite liver stages. Cell Rep..

[27] Doolan DL, Hoffman SL (1999). IL-12 and NK cells are required for antigen-specific adaptive immunity against malaria initiated by CD8^+^ T cells in the *Plasmodium yoelii* model. J Immunol..

[28] Narni-Mancinelli E, Chaix J, Fenis A, Kerdiles YM, Yessaad N, Reynders A (2011). Fate mapping analysis of lymphoid cells expressing the NKp46 cell surface receptor. Proc Natl Acad Sci USA.

[29] Nabekura T, Lanier LL (2016). Tracking the fate of antigen-specific versus cytokine-activated natural killer cells after cytomegalovirus infection. J Exp Med.

[30] Grivennikov SI, Tumanov AV, Liepinsh DJ, Kruglov AA, Marakusha BI, Shakhov AN (2005). Distinct and nonredundant in vivo functions of TNF produced by t cells and macrophages/neutrophils: protective and deleterious effects. Immunity.

[31] Zhang J, Marotel M, Fauteux-Daniel S, Mathieu AL, Viel S, Marcais A (2018). T-bet and Eomes govern differentiation and function of mouse and human NK cells and ILC1. Eur J Immunol.

[32] Chou C, Li MO (2018). Re(de)fining innate lymphocyte lineages in the face of cancer. Cancer Immunol Res.

[33] Ashley EA, Pyae Phyo A, Woodrow CJ (2018). Malaria. Lancet..

[34] Orago AS, Facer CA (1991). Cytotoxicity of human natural killer (NK) cell subsets for *Plasmodium falciparum* erythrocytic schizonts: stimulation by cytokines and inhibition by neomycin. Clin Exp Immunol.

[35] Hart GT, Tran TM, Theorell J, Schlums H, Arora G, Rajagopalan S (2019). Adaptive NK cells in people exposed to *Plasmodium falciparum* correlate with protection from malaria. J Exp Med.

[36] Deloron P, Chougnet C, Lepers JP, Tallet S, Coulanges P (1991). Protective value of elevated levels of gamma interferon in serum against exoerythrocytic stages of *Plasmodium falciparum*. J Clin Microbiol.

[37] Artavanis-Tsakonas K, Riley EM (2002). Innate immune response to malaria: rapid induction of IFN-gamma from human NK cells by live *Plasmodium falciparum*-infected erythrocytes. J Immunol..

[38] Luty AJ, Lell B, Schmidt-Ott R, Lehman LG, Luckner D, Greve B (1999). Interferon-gamma responses are associated with resistance to reinfection with *Plasmodium falciparum* in young African children. J Infect Dis.

[39] Mohan K, Moulin P, Stevenson MM (1997). Natural killer cell cytokine production, not cytotoxicity, contributes to resistance against blood-stage *Plasmodium chabaudi* AS infection. J Immunol..

[40] Choudhury HR, Sheikh NA, Bancroft GJ, Katz DR, De Souza JB (2000). Early nonspecific immune responses and immunity to blood-stage nonlethal *Plasmodium yoelii* malaria. Infect Immun.

[41] Korbel DS, Newman KC, Almeida CR, Davis DM, Riley EM (2005). Heterogeneous human NK cell responses to *Plasmodium falciparum*-infected erythrocytes. J Immunol..

[42] Artavanis-Tsakonas K, Eleme K, McQueen KL, Cheng NW, Parham P, Davis DM (2003). Activation of a subset of human NK cells upon contact with *Plasmodium falciparum*-infected erythrocytes. J Immunol..

[43] Baratin M, Roetynck S, Lépolard C, Falk C, Sawadogo S, Uematsu S (2005). Natural killer cell and macrophage cooperation in MyD88-dependent innate responses to *Plasmodium falciparum*. Proc Natl Acad Sci USA.

[44] Newman KC, Korbel DS, Hafalla JC, Riley EM (2006). Cross-talk with myeloid accessory cells regulates human natural killer cell interferon-gamma responses to malaria. PLoS Pathog.

[45] Mavoungou E, Luty AJ, Kremsner PG (2003). Natural killer (NK) cell-mediated cytolysis of *Plasmodium falciparum*-infected human red blood cells in vitro. Eur Cytokine Netw.

[46] Mavoungou E, Held J, Mewono L, Kremsner PG (2007). A Duffy binding-like domain is involved in the NKp30-mediated recognition of *Plasmodium falciparum*-parasitized erythrocytes by natural killer cells. J Infect Dis.

[47] Bouyou-Akotet MK, Issifou S, Meye JF, Kombila M, Ngou-Milama E, Luty AJ (2004). Depressed natural killer cell cytotoxicity against *Plasmodium falciparum*-infected erythrocytes during first pregnancies. Clin Infect Dis.

[48] Vaughan AM, Kappe SH, Ploss A, Mikolajczak SA (2012). Development of humanized mouse models to study human malaria parasite infection. Future Microbiol..

[49] Chen Q, Amaladoss A, Ye W, Liu M, Dummler S, Kong F (2014). Human natural killer cells control *Plasmodium falciparum* infection by eliminating infected red blood cells. Proc Natl Acad Sci USA.

[50] Saito F, Hirayasu K, Satoh T, Wang CW, Lusingu J, Arimori T (2017). Immune evasion of *Plasmodium falciparum* by RIFIN via inhibitory receptors. Nature.

[51] Ye W, Chew M, Hou J, Lai F, Leopold SJ, Loo HL (2018). Microvesicles from malaria-infected red blood cells activate natural killer cells via MDA5 pathway. PLoS Pathog.

[52] Arora G, Hart GT, Manzella-Lapeira J, Doritchamou JY, Narum DL, Thomas LM (2018). NK cells inhibit *Plasmodium falciparum* growth in red blood cells via antibody-dependent cellular cytotoxicity. eLife..

[53] Lo M, Kim HS, Tong RK, Bainbridge TW, Vernes JM, Zhang Y (2017). Effector-attenuating substitutions that maintain antibody stability and reduce toxicity in mice. J Biol Chem.

[54] Lopez-Verges S, Milush JM, Schwartz BS, Pando MJ, Jarjoura J, York VA (2011). Expansion of a unique CD57(+)NKG2Chi natural killer cell subset during acute human cytomegalovirus infection. Proc Natl Acad Sci USA.

[55] Schlums H, Cichocki F, Tesi B, Theorell J, Beziat V, Holmes TD (2015). Cytomegalovirus infection drives adaptive epigenetic diversification of NK cells with altered signaling and effector function. Immunity.

[56] Lee J, Zhang T, Hwang I, Kim A, Nitschke L, Kim M (2015). Epigenetic modification and antibody-dependent expansion of memory-like NK cells in human cytomegalovirus-infected individuals. Immunity.

[57] DiLillo DJ, Ravetch JV (2015). Differential Fc-receptor engagement drives an anti-tumor vaccinal effect. Cell.

[58] Uchida J, Hamaguchi Y, Oliver JA, Ravetch JV, Poe JC, Haas KM (2004). The innate mononuclear phagocyte network depletes B lymphocytes through Fc receptor-dependent mechanisms during anti-CD20 antibody immunotherapy. J Exp Med.

[59] Langhorne J, Quin SJ, Sanni LA (2002). Mouse models of blood-stage malaria infections: immune responses and cytokines involved in protection and pathology. Chem Immunol.

[60] Kim CC, Parikh S, Sun JC, Myrick A, Lanier LL, Rosenthal PJ (2008). Experimental malaria infection triggers early expansion of natural killer cells. Infect Immun.

[61] Kitaguchi T, Nagoya M, Amano T, Suzuki M, Minami M (1996). Analysis of roles of natural killer cells in defense against *Plasmodium chabaudi* in mice. Parasitol Res.

[62] Weidanz WP, LaFleur G, Brown A, Burns JM, Gramaglia I, van der Heyde HC (2010). Gammadelta T cells but not NK cells are essential for cell-mediated immunity against *Plasmodium chabaudi* malaria. Infect Immun.

[63] Who: World malaria report 2016. 2016.

[64] Carter JA, Mung’ala-Odera V, Neville BGR, Murira G, Mturi N, Musumba C (2005). Persistent neurocognitive impairments associated with severe falciparum malaria in Kenyan children. J Neurol Neurosurg Psychiatry.

[65] John CC, Panoskaltsis-Mortari A, Opoka RO, Park GS, Orchard PJ, Jurek AM (2008). Cerebrospinal fluid cytokine levels and cognitive impairment in cerebral malaria. Am J Trop Med Hyg.

[66] Kraemer SM, Smith JD (2006). A family affair: var genes, PfEMP1 binding, and malaria disease. Curr Opin Microbiol.

[67] Turner L, Lavstsen T, Berger SS, Wang CW, Petersen JE, Avril M (2013). Severe malaria is associated with parasite binding to endothelial protein C receptor. Nature.

[68] Hora R, Kapoor P, Thind KK, Mishra PC (2016). Cerebral malaria–clinical manifestations and pathogenesis. Metab Brain Dis.

[69] Storm J, Craig AG (2014). Pathogenesis of cerebral malaria–inflammation and cytoadherence. Front Cell Infect Microbiol..

[70] Taylor TE, Fu WJ, Carr RA, Whitten RO, Mueller JS, Fosiko NG (2004). Differentiating the pathologies of cerebral malaria by postmortem parasite counts. Nat Med.

[71] Stach JL, Dufrenoy E, Roffi J, Bach MA (1986). T-cell subsets and natural killer activity in *Plasmodium falciparum*-infected children. Clin Immunol Immunopathol.

[72] Epardaud M, Elpek KG, Rubinstein MP, Yonekura A-R, Bellemare-Pelletier A, Bronson R (2008). Interleukin-15/interleukin-15R alpha complexes promote destruction of established tumors by reviving tumor-resident CD8^+^ T cells. Cancer Res..

[73] Hamilton SE, Schenkel JM, Akue AD, Jameson SC (2010). IL-2 complex treatment can protect naive mice from bacterial and viral infection. J Immunol..

[74] Huenecke S, Zimmermann SY, Kloess S, Esser R, Brinkmann A, Tramsen L (2010). IL-2-driven regulation of NK cell receptors with regard to the distribution of CD16^+^ and CD16^−^ subpopulations and in vivo influence after haploidentical NK cell infusion. J Immunother.

[75] Swanson PA, Hart GT, Russo MV, Nayak D, Yazew T, Pena M (2016). CD8^+^ T cells induce fatal brainstem pathology during cerebral malaria via luminal antigen-specific engagement of brain vasculature. PLoS Pathog.

[76] Penet MF, Viola A, Confort-Gouny S, Le Fur Y, Duhamel G, Kober F (2005). Imaging experimental cerebral malaria in vivo: significant role of ischemic brain edema. J Neurosci.

[77] Potchen MJ, Kampondeni SD, Seydel KB, Birbeck GL, Hammond CA, Bradley WG (2012). Acute brain MRI findings in 120 Malawian children with cerebral malaria: new insights into an ancient disease. AJNR Am J Neuroradiol.

[78] Belnoue E, Kayibanda M, Vigario AM, Deschemin J-C, van Rooijen N, Viguier M (2002). On the pathogenic role of brain-sequestered alphabeta CD8^+^ T cells in experimental cerebral malaria. J Immunol..

[79] Nitcheu J, Bonduelle O, Combadiere C, Tefit M, Seilhean D, Mazier D (2003). Perforin-dependent brain-infiltrating cytotoxic CD8^+^ T lymphocytes mediate experimental cerebral malaria pathogenesis. J Immunol..

[80] Yañez DM, Manning DD, Cooley AJ, Weidanz WP, van der Heyde HC (1996). Participation of lymphocyte subpopulations in the pathogenesis of experimental murine cerebral malaria. J Immunol..

[81] Grau GE, Heremans H, Piguet PF, Pointaire P, Lambert PH, Billiau A (1989). Monoclonal antibody against interferon gamma can prevent experimental cerebral malaria and its associated overproduction of tumor necrosis factor. Proc Natl Acad Sci USA.

[82] Villegas-Mendez A, Greig R, Shaw TN, de Souza JB, Gwyer Findlay E, Stumhofer JS (2012). IFN-γ-producing CD4^+^ T cells promote experimental cerebral malaria by modulating CD8^+^ T cell accumulation within the brain. J Immunol..

[83] Hansen DS, Bernard NJ, Nie CQ, Schofield L (2007). NK cells stimulate recruitment of CXCR84+ T cells to the brain during *Plasmodium berghei*-mediated cerebral malaria. J Immunol..

[84] Ryg-Cornejo V, Nie CQ, Bernard NJ, Lundie RJ, Evans KJ, Crabb BS (2013). NK cells and conventional dendritic cells engage in reciprocal activation for the induction of inflammatory responses during *Plasmodium berghei* ANKA infection. Immunobiol..

[85] Hansen DS, Evans KJ, D’Ombrain MC, Bernard NJ, Sexton AC, Buckingham L (2005). The natural killer complex regulates severe malarial pathogenesis and influences acquired immune responses to *Plasmodium berghei* ANKA. Infect Immun.

[86] Hansen DS, Ryg-Cornejo V, Ioannidis LJ, Chiu CY, Ly A, Nie CQ (2014). The contribution of natural killer complex loci to the development of experimental cerebral malaria. PLoS ONE.

[87] Abt MC, Lewis BB, Caballero S, Xiong H, Carter RA, Susac B (2015). Innate immune defenses mediated by two ILC subsets are critical for protection against acute *Clostridium difficile* infection. Cell Host Microbe.

[88] Maroof A, Beattie L, Zubairi S, Svensson M, Stager S, Kaye PM (2008). Posttranscriptional regulation of II10 gene expression allows natural killer cells to express immunoregulatory function. Immunity.

[89] Fried M, Duffy PE (2015). Designing a VAR2CSA-based vaccine to prevent placental malaria. Vaccine..

[90] Salanti A, Staalsoe T, Lavstsen T, Jensen AT, Sowa MP, Arnot DE (2003). Selective upregulation of a single distinctly structured var gene in chondroitin sulphate A-adhering *Plasmodium falciparum* involved in pregnancy-associated malaria. Mol Microbiol.

[91] Salanti A, Dahlback M, Turner L, Nielsen MA, Barfod L, Magistrado P (2004). Evidence for the involvement of VAR2CSA in pregnancy-associated malaria. J Exp Med.

[92] Sartelet H, Schleiermacher D, Le-Hesran JY, Graesslin O, Gaillard D, Fe M (2005). Less HLA-G expression in *Plasmodium falciparum*-infected third trimester placentas is associated with more natural killer cells. Placenta.

[93] Ordi J, Menendez C, Ismail MR, Ventura PJ, Palacin A, Kahigwa E (2001). Placental malaria is associated with cell-mediated inflammatory responses with selective absence of natural killer cells. J Infect Dis.

[94] Wallace AE, Fraser R, Cartwright JE (2012). Extravillous trophoblast and decidual natural killer cells: a remodelling partnership. Hum Reprod Update..

[95] Othoro C, Moore JM, Wannemuehler KA, Moses S, Lal A, Otieno J (2008). Elevated gamma interferon-producing NK cells, CD45RO memory-like T cells, and CD4 T cells are associated with protection against malaria infection in pregnancy. Infect Immun.

[96] Perkins DJ, Were T, Davenport GC, Kempaiah P, Hittner JB, Ong’echa JM (2011). Severe malarial anemia: innate immunity and pathogenesis. Int J Biol Sci..

[97] Munde EO, Okeyo WA, Raballah E, Anyona SB, Were T, Ong’echa JM (2017). Association between Fcgamma receptor IIA, IIIA and IIIB genetic polymorphisms and susceptibility to severe malaria anemia in children in western Kenya. BMC Infect Dis.

